# The Hospital

**Published:** 1918-01-05

**Authors:** 


### THE INSTITUTIONAL WORKER

**Being a Special Supplement to "The Hospital"**

### OUR BUREAU OF INFORMATION.

**Rules for Correspondents.**

1. Every letter must be accompanied by the coupon to be cut from the back cover (inside page) of The Hospital, current issue, and must contain the name and address of the correspondent with pseudonym for publication if desired. Replies by post cannot be given save under exceptional circumstances at the Editor's discretion.

2. Letters from Approved Homes in reply to special needs published in the Bureau should state terms and full particulars, and be sent prepaid under cover to the Editor of the Bureau with name written across coupon for identification.

3. Proprietors of Homes which have not yet been entered on the List of Approved Homes, but have spare accommodation likely to suit special needs, are invited to write for an application form for registration. The fee for registration, which includes two announcements of the Home in the Bureau and other privileges, is 10s.

4. All communications to be addressed to the Editor of The Hospital, 28 Southampton Street, Strand, London, W.C. 2, and marked "Bureau of Information."

### INSTITUTION FACTS AND FIGURES.

### How to Organise a Mothers' and Babies' Welcome.

#### Answer to November Question.

By L. L. A.

The question was:

**Outline a simple scheme which a midwife working independently and single-handed in a scattered country district could adopt to start a "Mothers and Babies' Welcome."**

In the district where I live the conditions are similar to those mentioned in the above question, and the midwife has to do everything single-handed. Nevertheless she runs a weekly "Mothers' and Babies' Welcome" which bids fair to be a very successful innovation, and I see no reason why her example could not easily be followed by others similarly circumstanced.

Even in these times of almost universal war-work for women there can generally be found three or four motherly—and preferably *married*—women who, if approached on the matter, would be only too glad to help voluntarily at the weekly "Welcome."

The midwife should take care to choose women who have the confidence and affection of their neighbours, for it will only be detrimental to the success of the "Welcome" if she chooses high-handed and unpopular helpers—no matter how extensive their knowledge or superior their station. Given the right sort of women, it will be comparatively easy to get the mothers to attend, especially if no trouble be spared to make the meetings bright and interesting. Country life is, as a rule, so monotonous that the inhabitants generally welcome any opportunity afforded for social intercourse, and it is thus as a rule easier to make the "Welcome" a success than it is in the town, where there are so many other attractions and diversions.

It is no use attempting to run a "Welcome" *every day*, especially in a scattered district. It is far better to have a well-attended weekly or bi-weekly meeting than frequent badly attended meetings. Moreover, the midwife will not have time to devote more than one or two afternoons to them, and if they are to be successful, no matter how efficient her helpers, she must be in supreme charge on such occasions.

If funds are limited, the midwife would not be able to rent a room for the meetings, but if money is fairly plentiful there should be a special room, well lighted and heated, kept for the "Welcome" alone. Failing this, the church or village hall might be available—or one of the voluntary helpers might be able to furnish the necessary accommodation. The meetings should take the form of a simple address and chat—and except where she has unusually gifted helpers with a practical knowledge of baby welfare, the midwife will have to conduct these herself. The subject matter should be made as interesting as possible—diagrams and illustrations should be used, and the mothers should be encouraged to ask questions and bring their special difficulties for solution. If there were two rooms, it would be advisable for all except the very youngest babies to be quartered there with one of the helpers, and this room should be fitted up with two or three cots for the sleepy babies and well provided with toys and games. This ensures a quiet lecture, and gives the mother opportunity to have a rest and to give her undivided attention to the subject. If one of the helpers could give an occasional talk on the making of baby garments it would be a great advantage, and such a class is generally a great favourite with the mothers.

At the close of the lecture each child should be weighed, and weekly records should be kept in a separate book for each baby, so that it may be seen if he is making progress or falling off in health. The helpers will soon be efficient enough to relieve the midwife of most of the routine work.

A supply of the best foods, such as Glaxo, Virol, etc., should be kept at the "Welcome" and retailed to the mothers at specially reduced prices.

At the close of the proceedings it will generally be advisable to entertain the mothers to tea, especially when they have had to come a long distance—although, of course, in these abnormal times it will generally be found necessary to make a small charge. Once or twice a year a mothers' and babies' social should be held, and this might well be combined with a baby show, when prizes could be given for the best babies.

I have only given the general lines on which a "Welcome" could be started. Once this is in working order it will be a simple matter to attempt to organise something on a larger scale. The great thing is to first of all obtain the co-operation of the mothers—the rest will follow and be comparatively easy.

**The Editor will be glad to receive correspondence and to consider contributions upon all subjects relating to institutional work which affect the welfare of institutional workers.**

### JANUARY QUESTIONS.

**1. Extending a Hospital Engineer's Work.**

In what ways can a hospital engineer be useful in addition to his routine work of attending to the heating apparatus?

**2. A Domestic Matter in a Nurses' Home.**

Describe the cleanest, quickest, and most careful method of washing and drying crockery in a nurses' home to accommodate 180 persons.

### RULES.

The following rules must be observed:—

1. Contributions must be written on one side of the paper. Brevity and terseness are desirable features. The MSS. must bear the name and address of the sender and be accompanied by coupon to be cut from the back cover (inside page) of the current issue of The Hospital. A pseudonym must be chosen if the name is not to be published.
2. Contributions must be addressed to the Editor of The Hospital, Institutional Worker Supplement, 28 & 29 Southampton Street, Strand, London, W.C. 2, should reach him before the end of the current month, and be marked in the left-hand corner "Facts and Figures."

**A minimum payment of Five Shillings will be made for each published answer to the January Questions.**

### QUESTIONS INVITED.

In connection with our Question Box, we offer every month five shillings each for the two best questions which are sent in for consideration. The questions may be on any institutional subject and concern any department, from the secretary's to the porter's, or the matron's to the domestic staff; the one essential is that they must be practical and deal with points that have a definite relation to hospital or institutional administration, upkeep, finance, or management. Points relating to artificers' work, housekeeping, the laundry, out-patients, and other practical matters will be welcome. It is hoped that thus, when difficult or doubtful points arise in the course of their work, institutional workers will be encouraged to put them in the form of a question and send them to the Editor, The Hospital Bureau of Information, 28 Southampton Street, Strand, W.C. 2, marked "Question Box."

The best questions will be published in due course and our readers will be given the opportunity of answering them. Thus all institutional workers may be able to co-operate with the view of helping the work and smoothing the difficulties of each other. In short, we want the Question Box of the *Institutional Worker* to be freely used by every worker habitually.

### ENQUIRIES AND ANSWERS.

### *THE SICK AND IN NEED.*

### Case of Tuberculous Glands of Neck.

If the case is one which the doctor considers requires some surgical treatment, then we suggest that you apply for admission to one of the London general hospitals, such as, for instance, the Middlesex Hospital, Mortimer Street, W.; or St. Bartholomew's Hospital, West Smithfield, London, E.C. Otherwise it is possible that the case might be received at the Hospital for Consumption and Diseases of the Chest, Brompton, S.W. A coupon should be enclosed with each inquiry.—B. T. C.

### Home for Incurables.

The case you are interested in might possibly be admitted to the St. Cyprian's Home for Incurables, 31 The Grove, Hammersmith, W. Applications should be made to the Hon. Secretary. A small weekly payment is required. Failing that, you might apply to St. Joseph's Hospital for Incurables, Burlington Lane, Chiswick. Admission is by selection and a weekly payment in advance.—Joyce.

### Scottish Hospital for Incurables.

Patients who require such constant attention and dressing as cannot adequately be given at home are received at the Royal Edinburgh Hospital for Incurables, Edinburgh. Application should be made to the Secretary in the first place.—Jean.

### Pay Hospital for Women.

The case is one for whom you might seek admission to the Grosvenor Hospital for Women, Vincent Square, Westminster, S.W. There are a limited number of private wards, for which a weekly payment of £2 2s. is required.—P. S. L.

### Dental Treatment.

If you are unable to pay for the services of a dental surgeon, your best course will be to apply for treatment at the Royal Dental Hospital of London, Leicester Square. There is also an excellent dental department at Guy's Hospital.—Wee Macgregor.

### Hospitals for Women.

We suggest that you apply to one of the following institutions: Samaritan Free Hospital for Women, Marylebone Road, W.; or Chelsea Hospital for Women, Fulham Road, S.W. Both are excellent institutions.—District.

### *MISCELLANEOUS.*

### A Reliable Water-Softener.

Particulars and diagram of the Paterson Horizontal "B" Type Cold-process Water-softener, as used at the Ham Green Hospital and Sanatorium, Bristol, appeared in The Hospital, published November 4, 1916, under the heading "The Heating of Hospitals."—Secretary.

### Fuel Economies.

There are many simple ways of economising in the use of fuel—for instance, if a fireplace happens to be larger than necessary and only a small, slow-burning fire is required, a part of the space may be filled with balls made by mixing coal with clay. These, when dry, will, if placed in the fire, form a glowing mass and be consumed very slowly. Small coal and slack accumulated in the cellar should be used for damping down fires or for making the coal balls referred to. Another important point to remember is that the ashes in the grate should be carefully screened and made use of.—Economist.

### Operating Theatre Light.

Probably one of the best types of operating theatre lights is that manufactured by the Scanlan-Morris Company, of Madison, Wisconsin, U.S.A. It is known as the "Bartlett Noshado Lite." The reflectors are arranged to focus all light rays on an area of 18 inches, the fixture being constructed so that the rays from the individual lights meet at an angle of 45 degrees, thus making possible the examination of deep wounds. The fixture consists of a ring 6 feet in diameter suspended from the ceiling plate by an adjustable ball joint. The ring is fitted at intervals with eight parabolic reflectors.—L. A. C.

### *EMPLOYMENT AND TRAINING.*

### Training in General Nursing.

You could obtain a three years' course of general training in the wards of the Gloucester Royal Infirmary. Applicant must be between twenty-two and thirty years of age and must undertake to remain in the service of the infirmary for a further period of one year.—A. T. N.

### Medical Schools for Women.

The fees for the complete course of study at the London (Royal Free Hospital) School of Medicine for Women, 8 Hunter Street, Brunswick Place, W.C., range from £140 to £160, in accordance with the diplomas or degrees worked for.—Medicine.

### Training in Massage and Electricity.

The best means of obtaining training in massage and electricity is in the wards of a hospital, and we suggest that you write to the following institutions for particulars of their training: The National Hospital, Queen Square, Bloomsbury, W.C., and the West-End Hospital, 73 Welbeck Street, London, W. Both these institutions are open to outside students.—Silvester.

